# The emerging role of subchondral bone homeostasis in osteoarthritis

**DOI:** 10.1016/j.jot.2026.101233

**Published:** 2026-10-13

**Authors:** Antonia Donat, Weixin Xie, Assil-Ramin Alimy, Anke Baranowsky, Tim Rolvien, Johannes Keller

**Affiliations:** a Musculoskeletal University Center Munich, Ludwig-Maximilians-University Munich (LMU), Marchioninistr. 15, Munich, 81377, Germany; b Department of Trauma and Orthopaedic Surgery, University Medical Center Hamburg-Eppendorf, Martinistr. 52, Hamburg, 20246, Germany

**Keywords:** Osteoarthritis, Crosstalk, Joint disease, PIEZO1, RANKL, Subchondral bone

## Abstract

The treatment of progressive articular cartilage degeneration and consequent joint deterioration, also known as osteoarthritis (OA), remains a major challenge in musculoskeletal medicine. The subchondral bone has become a key area of research, as early alterations in bone remodelling and structure may precede cartilage degeneration and contribute to disease initiation and progression. Therefore, this review aims to summarise our current understanding of the role of subchondral bone in the initiation and progression of OA. Scientific databases were analysed, focusing on studies published within the last 15 years. Both preclinical *in vitro* and *in vivo* studies, as well as clinical trials, were included. Morphological changes of the subchondral bone in OA – primarily driven by abnormal bone remodelling – have been thoroughly characterised within the last years. However, the underlying cellular and molecular mechanisms remain only partially elucidated. As increasing evidence suggests that metabolically and mechanically driven changes in subchondral bone homeostasis, including signalling pathways such as RANK/RANKL/OPG, PIEZO1, and MMP13/TGF-β, may actively contribute to OA progression, this review discusses how this new understanding may influence current and future therapeutic strategies. Furthermore, we critically assess whether anti-osteoporotic agents may represent disease-modifying strategies in OA by targeting subchondral bone remodelling.

**The Translational Potential of this Article**: This review provides an update on subchondral bone as a potential contributor to OA progression and discusses how mechanistic insights from preclinical studies can lead to clinical therapeutic opportunities, emphasising bone remodelling, mechanosensing, angiogenesis and neural invasion. Furthermore, selective molecular targets for the development of disease-modifying treatments and the rationale for repurposing anti-osteoporotic drugs to translate bone-targeted strategies into clinical OA management are evaluated.

## Graphical abstract

**Figure:**
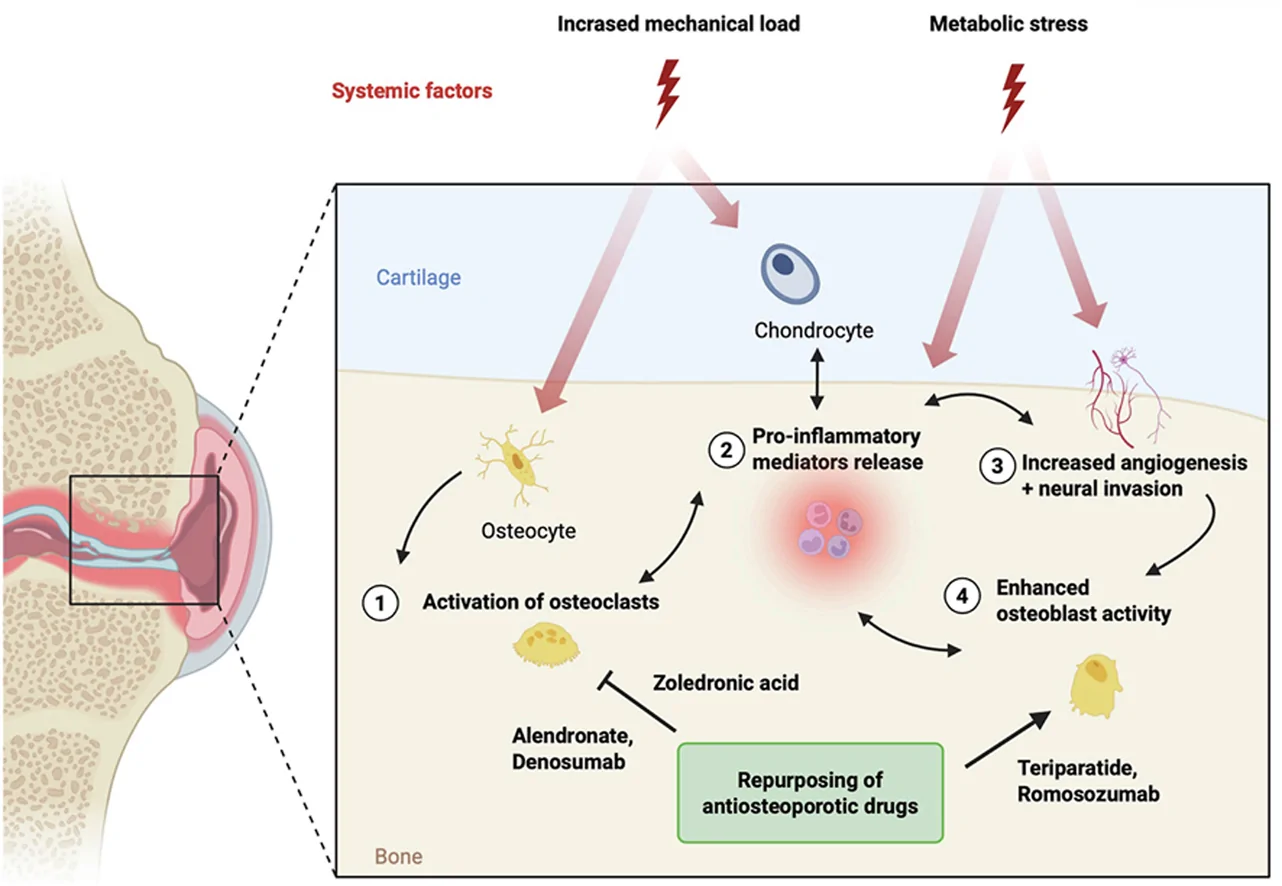

## 1 Introduction

Osteoarthritis (OA) is a progressive whole-joint disease characterised by joint degeneration, typically arising from increased mechanical loading or as a sequela of joint injury. Its main characteristics include degradation of the articular cartilage, synovial inflammation, and profound structural alterations in the subchondral bone. These tissues interact through complex biomechanical and biochemical signalling pathways, which contribute to inflammation, structural remodelling, and joint dysfunction. Thus, OA reflects a breakdown of the joint as a whole organ system rather than a disorder confined to cartilage alone. In 2019, more than 500 million people were affected by OA globally, making it one of the most prevalent and burdensome musculoskeletal disorders. Global epidemiological data show a continuous rise in OA prevalence across all age groups, with particularly strong increases in knee and hip OA associated with aging populations [1]. As there is currently no cure, a detailed understanding of pathological mechanisms driving OA initiation and progression is of utmost importance.

As early as 1999, the subchondral bone was identified as a potential therapeutic target for a pharmacological approach to treating OA [2]. This assertion was based on the recognition that progression to advanced joint damage and pain appears to depend not only on cartilage decay, but also on the responses in the subchondral bone. Since then, drugs originally developed for the treatment of osteoporosis have been repurposed in clinical OA studies, including strontium ranelate and bisphosphonates. In recent years, growing evidence has suggested that subchondral bone alterations do not merely follow cartilage loss, but may even precede cartilage deterioration. Clinical imaging and experimental studies indicate that subchondral bone remodelling - characterised by altered bone turnover, mineralisation, and microarchitecture - occurs at very early stages of OA [3,4]. At the cellular level, altered subchondral bone turnover reflects an imbalance between osteoclast-mediated resorption and osteoblast-driven bone formation, processes that are tightly orchestrated by osteocytes as embedded mechanosensors. Dysregulation of this coupled remodelling system can shift subchondral bone structure and mechanics, with downstream consequences for the overlying cartilage and overall joint integrity. Accumulating evidence from clinical and preclinical studies suggests that specifically altered osteocyte signalling contributes to abnormal remodelling, disease progression and pain [5,6]. In parallel, there is increasing recognition of “crosstalk” between subchondral bone cells and chondrocytes in the overlying cartilage [7], which may occur differentially depending on disease stage, joint type, and local biomechanical environment (Supplementary Table 1).

Given this rich and evolving picture, this review aims to provide an update on the latest insights on the role of subchondral bone in the initiation and progression of OA, distinguishing between early versus late disease stages and highlighting areas where mechanistic understanding remains incomplete. Emphasis is placed on integrating structural, molecular, and therapeutic evidence into a stage- and endotype-informed framework that reflects the current understanding of subchondral bone biology in OA.

## 2 Methods

A narrative review was conducted to summarise and contextualise current evidence published between January 01, 2010 and July 31, 2026. The electronic scientific databases PubMed/MEDLINE, Scopus, and Web of Science were searched to identify relevant literature. The search strategy comprised keywords related to the core topic combined with Boolean operators (AND/OR). Search strings included “osteoarthritis” AND “subchondral bone”, “subchondral bone remodelling” AND “cartilage degeneration”, “osteocytes” OR “chondrocytes” AND “mechanotransduction” and “antiosteoporotic drugs” AND “osteoarthritis”. Eligible studies comprised original research articles encompassing preclinical *in vitro* experiments, *in vivo* animal studies, and human clinical studies or trials that contributed mechanistic, translational, or therapeutic insights relevant to the review topic. Publications were limited to peer-reviewed articles in English to ensure scientific rigor and interpretability. Studies were excluded if they were not directly relevant to the defined scope of the review, lacked primary data (e.g., editorials, opinion pieces, conference abstracts without full data), or focused on unrelated outcomes. Case reports and small case series were excluded unless they provided unique mechanistic insights. Relevant references were then prioritised based on (1) relevance to the central theme, (2) methodological rigor and (3) scientific contribution, guided by the authors’ expertise in OA and subchondral bone biology. Thematic organisation of the literature was primarily guided by pathophysiological processes and studies thus grouped into themes including microstructural bone changes, osteoblast, osteoclast and/or osteocyte signalling, angiogenesis, sensory nerve innervation and therapeutic approaches. Reference lists of selected articles were also screened to identify additional relevant publications. Although the review focuses on recent evidence, essential publications published before 2010 were selectively included for the sake of completeness. Due to the descriptive nature of this review, no predefined protocol or quantitative evidence synthesis was performed.

## 3 Analysis of subchondral bone morphology highlights stage-, region-, and endotype-specific patterns

The subchondral bone is defined as the bone beneath the calcified cartilage within synovial joints. Anatomically, it is divided into two distinct structural components: the subchondral bone plate, characterised as the compact, cortical bone-like layer located directly beneath the calcified cartilage; and the underlying subchondral trabecular bone, which forms part of the load-transmitting interface of the joint and exhibits higher porosity than the subchondral bone plate. The thin layer at the transition from calcified to non-calcified cartilage is known as the tidemark, which forms part of the osteochondral interface [8] (Fig. 1). Imaging of the subchondral bone in OA - including magnetic resonance imaging (MRI)-based assessment of bone marrow lesions [9], and advanced computed tomography (CT)- and μCT-based approaches [10] - has recently been comprehensively reviewed elsewhere. Therefore, these imaging modalities are not covered in detail in the present review, which instead focuses on the biological and therapeutic implications of subchondral bone remodelling.

**Fig. 1:**
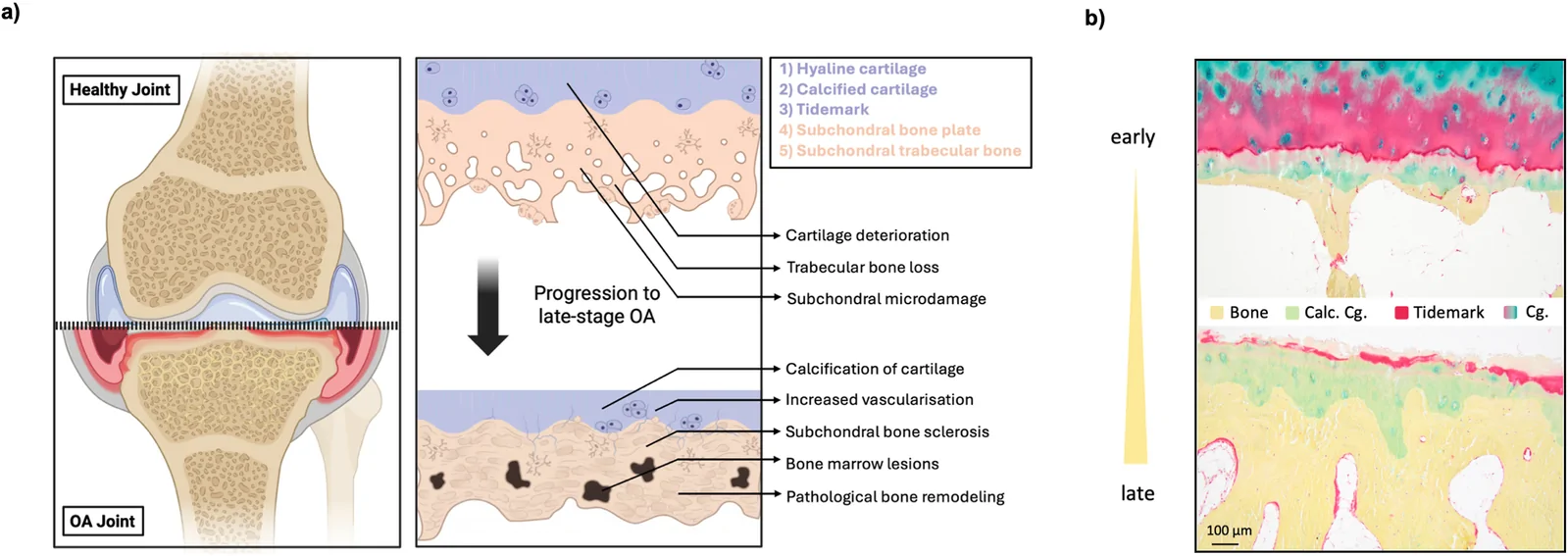
Schematic illustration (a) and histological images of Movat-Pentachrome-stained sections (b) of the osteochondral unit and respective changes in early and late-stage osteoarthritis.

OA development and progression are distinct biological processes, characterised by stage- and region-specific alterations in subchondral bone remodelling. Early stages of OA are generally characterised by a predominantly high-turnover, osteoclast-driven resorptive phenotype, resulting in an increase in osteoclastic bone resorption of the subchondral bone plate with subsequent subchondral osteopenia. Histological analyses have identified tidemark breaching as a common finding in asymptomatic chondropathy; however, tidemark thickening is typically not observed in early OA [11]. Initial cartilage damage with loss of proteoglycans, disruption of collagen structure and a shift from collagen type II to collagen type I has been described in early OA [12], and may occur upstream or in parallel with subchondral bone alterations. These changes are sometimes even accompanied by cartilage thickening in weight-bearing regions [13]. In contrast, late disease stages are generally characterised by a predominantly sclerotic, osteoblast-driven phenotype, with increased osteoblastic bone formation extending to the subchondral trabecular bone, leading to subchondral sclerosis [14]. Additionally, tidemark multiplication at the osteochondral junction is consistently observed in advanced disease [15]. Further hallmarks of late-stage OA include progressive cartilage damage with irreversible structural changes, such as deep erosions and reduced cellularity, leading to thinning of the cartilage and ultimately joint deformation caused by mechanical failure of subchondral bone [16,17]. These late-stage changes reflect a shift toward increased subchondral bone stiffness, which may alter load distribution and exacerbate subsequent complete cartilage breakdown (summarised in Table 1).

**Table 1:** Stage-specific characteristics of the subchondral bone and associated molecular mechanisms in OA.

OA stage	Subchondral bone phenotype	Dominant cell populations	Key signalling pathways	Preclinical evidence	Clinical evidence
**Early**	- Subchondral osteopenia - Increased bone resorption - Trabecular thinning - Tidemark breaching without thickening	Primarily: Osteoclasts Other: Osteocytes, MSC dysfunction	RANK/RANKL/OPG PIEZO1/YAP IGSF11 miRNA-21	Osteoclast-driven bone resorption leads to cartilage degeneration [18] PIEZO1 mechanosensing controls bone mass [19] Adipocyte-derived RANKL enhances osteoclastogenesis [20] IGSF11 stimulates osteoclast differentiation [21] miRNA-21 mediates RANKL-induced osteoclastogenesis [22]	Increased RANKL and osteoclasts correlate with cartilage loss [23] Early bone remodelling detected by imaging [3]
**Intermediate**	- Increased BV/TV - Early sclerosis - BMLs - Angiogenesis - Sensory innervation	Primarily: Mixed osteoclast/osteoblast activity Other: Type H vessels, sensory nerves	PI3K/AKT/OPN CXCL12 PDGF-BB/EP4 NGF, Netrin1	Type H vessels precede sclerosis [24] OPN induces angiogenesis and sclerosis [25] CXCL12 promotes endothelial migration [26] NGF blockade reduces pain [27]	BMLs associated with OA progression and pain [28] NGF signalling correlates with OA progression [29,30]
**Late**	- Subchondral bone sclerosis - Bone cysts /osteophytes - Tidemark multiplication - Increased bone stiffness	Primarily: Osteoblasts	TGF-β MMP13/PLR PGE2	Osteocyte TGF-β disruption causes sclerosis and cartilage degeneration [31] MMP13 deficiency disrupts bone matrix and cartilage [32]	Increased bone sclerosis and structural damage in imaging [15,33,34] Vascular perfusion correlates with pain [35]

Clinical and experimental evidence indicates that OA-related alterations in the subchondral bone are region-specific and closely linked to joint biomechanics. In the hip, femoral head samples from OA patients have shown morphometric variations that depend more on the anatomical site than on the distribution of cartilage lesions. In this regard, Taheri et al. reported that load-bearing regions exhibited increased trabecular thickness compared with non-load-bearing regions, independent of local cartilage damage [36], suggesting that mechanical loading exerts a primary influence on bone structure that might precede or occur independently of cartilage pathology. In general, femoral heads affected by primary OA exhibit increased osteoblast numbers and greater heterogeneity in matrix mineralisation compared to non-OA controls. These abnormalities are spatially associated with severe cartilage degeneration, underscoring the functional coupling between abnormal bone remodelling and cartilage breakdown [37]. Longitudinal clinical imaging studies have demonstrated progressive increases in subchondral trabecular bone volume fraction (bone volume per tissue volume, BV/TV) and trabecular thickness, accompanied by reduced trabecular separation, as joint-space narrowing advances [33,34]. Using multi-detector row computed tomography, Chiba et al. demonstrated that bone changes precede end-stage cartilage loss, while Kaneta et al. confirmed that increased BV/TV strongly correlates with radiographic OA severity and clinical symptoms. This supports an association but does not definitively establish a causal relationship between osseous alterations and subsequent cartilage decay. Complementing these findings, a recent study proposed three subchondral bone-defined endotypes in hip OA. These include I) low bone mass and low turnover; II) intermediate bone mass and high turnover; and III) high bone mass and high turnover, with cluster III also standing out as an osteocyte-rich endotype [38]. Importantly, these subchondral bone endotypes highlight that microstructural, cellular, and compositional changes in the subchondral bone should not necessarily be viewed as merely stage-specific, but may represent biologically distinct types of OA, which has several important implications. First, it indicates that radiographic OA severity (Kellgren–Lawrence grade) does not capture the underlying cellular and structural heterogeneity of the disease, which may explain why interventions selected on radiographic criteria alone have produced inconsistent results. Second, it positions osteocytes, rather than only osteoclasts or osteoblasts, as drivers of a distinct subchondral phenotype, suggesting that osteocyte-targeted strategies warrant exploration in selected patient subgroups. Third, the close link between bone marrow adipocyte morphology and osteoblast activity, independent of systemic body mass index (BMI) and bone mineral density (BMD), supports local metabolic crosstalk within the subchondral marrow as a candidate therapeutic axis. Together, these observations argue that future trials of bone-targeted disease-modifying OA drugs should prospectively stratify by subchondral endotype rather than by joint-level radiographic severity, while also accounting for disease stage, with antiresorptives most likely to benefit patients with high-turnover/resorptive phenotypes, particular during earlier OA stages, osteoanabolic agents potentially targeting selected low-turnover phenotypes, and emerging osteocyte-modulating strategies reserved for the osteocytic endotype.

In the osteoarthritic knee joint, mechanical alignment appears to be the major determinant of cartilage degeneration, pathological subchondral bone formation and osteocyte lacunocanalicular network alterations [39]. Han et al. also demonstrated that varus or valgus malalignment leads to asymmetric increases in bone volume and stiffness in overloaded compartments, which in turn are associated with faster cartilage loss [40]. Increased bone mass and altered trabecular architecture in weight-bearing subchondral regions correlate not only with cartilage damage but also with soft tissue lesions, such as meniscal degeneration, suggesting a shared mechanical aetiology [41]. Subchondral bone marrow lesions (BMLs) are likewise associated with malalignment and accelerated OA progression, as shown by MRI-based longitudinal analyses [28]. These lesions correspond to areas of high bone turnover and microdamage, reflecting localised remodelling responses to altered load distribution. Notably, subchondral bone cysts have also been identified as foci of increased bone remodelling associated with articular cartilage degeneration [42]. Evidence from animal models supports the notion that subchondral bone changes are not merely secondary to cartilage loss but can actively contribute to OA progression. In a murine OA model, for instance, increased osteoclast-mediated bone resorption in the subchondral compartment preceded cartilage degeneration [43].

Collectively, these findings indicate a dual role of subchondral bone in OA: first, as a tissue that dynamically responds to altered mechanical loading and cartilage damage; and second, as an active regulator of cartilage health, joint biomechanics and pain. However, the relative contribution of these roles appears to vary across disease stages, joint types, and OA subgroups (Fig. 2).

**Fig. 2:**
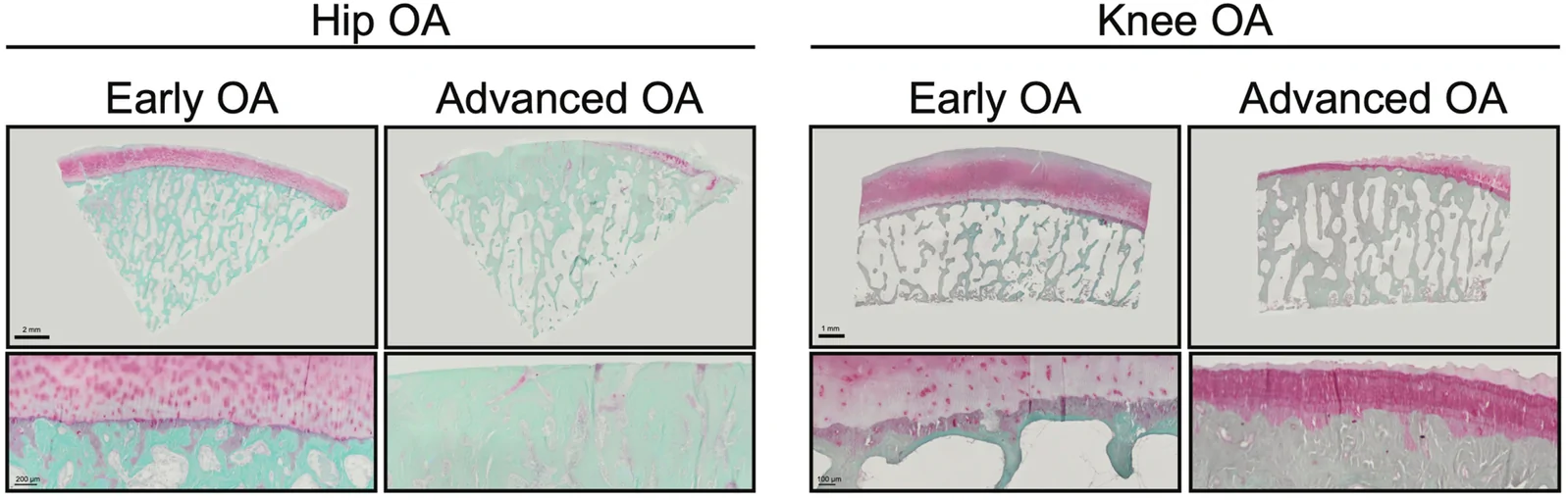
Comparison of stage- and joint-specific pathological alterations in human OA using representative Safranin O–stained histological sections.

## 4 Underlying molecular mechanisms

Establishing a unified mechanistic framework for subchondral bone alterations in OA remains challenging, as multiple, interdependent signalling pathways are activated concurrently, precluding a simple linear upstream–downstream cascade model of disease progression. Primary changes include abnormal mechanical loading and/or systemic metabolic stress, which are thought to precede and shape local molecular changes within the subchondral bone. Chondrocytes and osteocytes detect altered mechanical loading, leading to aberrant intracellular signalling, differential expression of auto- and paracrine or membrane-based signalling molecules, and subsequent pathological bone remodelling as described below. Concomitantly, local inflammatory mediators, lipid metabolites and hypoxia-related signals further amplify this initial mechanobiological response, further promoting osteoblast and osteoclast dysfunction. This disruption of subchondral bone remodelling promotes microarchitectural changes, subsequently accompanied by increased angiogenesis and sensory nerve ingrowth associated with enhanced pain perception. As the barrier function of the osteochondral junction is impaired, a pathological, bidirectional crosstalk between bone and cartilage is established, as comprehensively summarised elsewhere [7]. Importantly, these pathological changes evolve heterogeneously in a spatially and temporally progressive manner, with early focal alterations in subchondral bone remodelling often preceding, and potentially contributing to, joint-wide degeneration.

### 4.1 Metabolically driven changes

OA has traditionally been viewed as a primarily mechanically driven degenerative disease. However, accumulating evidence supports a complementary model in which metabolic dysregulation contributes to disease initiation and progression. Alterations within the subchondral bone microenvironment and systemic metabolic disturbances, including obesity and diabetes, both influence OA progression. On a cellular level, recent experimental evidence has shown that mesenchymal stem cells (MSCs) from OA knees exhibit impaired osteogenic differentiation, resulting in an increased proportion of bone marrow adipocytes during the course of OA progression [44]. Imaging studies, however, have not consistently captured these changes, as bone marrow fat distribution in the femoral head was reported to decline over the course of hip OA progression [45], highlighting potential differences between joint and study types. Further mechanistic studies showed that bone marrow adipocyte-derived RANKL enhances osteoclastogenesis [20,46], providing a possible mechanism underlying the catabolic bone metabolism observed in early OA. Additionally, lipolysis of bone marrow adipocytes has been implicated in the high bone remodelling phenotype observed in hand OA, as shown by a recent morphometric analysis [47]. This concept also potentially carries translational implications, as modulation of local adipocyte metabolism, particularly lipolytic pathways, may represent an alternative strategy to influence subchondral bone remodelling and potentially ameliorate OA progression. However, no pharmacological inhibitors of lipolysis have yet been clinically validated for OA treatment, and the causal relevance of these pathways in human disease remains to be established. More broadly, direct causal evidence linking altered mechanical loading to systemic or local metabolic dysregulation within subchondral bone remains sparse; most current data are correlational, and mechanistic studies establishing this directional link in OA are an open area of investigation.

Regarding systemic metabolic drivers, higher bodyweight increases mechanical loading, suggesting that many OA phenotypes are predominantly mechanically driven. However, rates of OA in non-weight bearing joints have also been found to correlate with body weight [48], indicating the contribution of systemic, load-independent mechanisms. The metabolic syndrome (e.g. obesity, insulin resistance, elevated blood pressure) is associated with chronic inflammation, hyperglycaemia and microvascular damage. In the subchondral bone, inflammatory signalling via interleukin-1β (IL-1β) has been shown to enhance osteogenic differentiation and subchondral bone glycolysis, leading to increased bone sclerosis in experimental models [49]. Inflammation furthermore leads to an increase in reactive oxygen species (ROS) due to mitochondrial dysfunction in chondrocytes, osteoblasts and osteocytes [50]. Accumulation of ROS can ultimately lead to chondrocyte senescence and apoptosis [51,52], and hence aggravate disease progression. Experimental OA data suggest that oxidative stress is associated with increased subchondral bone sclerosis in later disease stages [53]; however, robust clinical evidence specifically addressing ROS in subchondral bone in OA remains limited, and thus these findings remain largely associative.

On a systemic level, several signalling molecules are secreted by adipocytes, most notably adipokines such as leptin and adiponectin. Over the course of OA progression, adipokines modulate chondrocyte degeneration and apoptosis, inflammatory signalling and bone metabolism over complex molecular pathways, as recently summarised elsewhere [54,55]. An emerging field in research focuses on the role of adipokines in pain. Recent evidence suggests a role of leptin in pain generation, as lipodystrophic mice were protected from severe OA and pain after surgical destabilization of the medial meniscus [56]. In a clinical study, adiponectin serum levels correlated with pain scores in early to intermediate knee OA [57]. Nonetheless, further research is required to establish causal relationships between adipokines and pain perception.

Targeting the metabolic axis of OA yields a broad range of therapeutic implications. A recent placebo-controlled clinical trial investigated the use of glucagon-like peptide-1 (GLP-1) receptor agonist semaglutide in patients with obesity and mild to moderate knee OA. Here, reduced pain scores were observed in the treatment group [58]. However, it remains unclear whether the observed effects were secondary to weight loss, which was higher in the treatment group. Furthermore, no structural OA parameters, such as cartilage loss and subchondral bone alterations, were assessed. Notably, preclinical data showed that GLP-1 receptor activation reduced structural OA progression in a mouse model [59], suggesting a potential disease-modifying effect that requires validation in humans. In sum, metabolic dysregulation – both at the cellular and systemic level – represents an important contributor to OA pathogenesis, although its relative contribution varies across OA subtypes. While the metabolic axis remains an emerging field, it provides a broad perspective for future research and therapeutic approaches.

### 4.2 Mechanotransduction and osteocytic pathways

Mechanical cues are essential for maintaining cartilage homeostasis, as both chondrocytes and osteocytes continuously sense and respond to joint loading through mechanotransduction pathways. Although both chondrocytes and osteocytes contribute to mechanosensing, they fulfil complementary roles. Chondrocytes primarily detect changes within the articular cartilage matrix, whereas osteocytes integrate mechanical signals within the mineralized subchondral bone and coordinate adaptive bone remodelling through downstream regulation of osteoblast and osteoclast activity. Chondrocytes primarily detect compressive loading via membrane-spanning ion channels including Piezo-type mechanosensitive ion channel component 1 (PIEZO1), generating Ca^2+^-mediated catabolic responses within articular cartilage; osteocytes, embedded within the bone matrix, transduce strain through the lacunocanalicular network and transforming growth factor beta (TGF-β)-dependent perilacunar remodelling, regulating osteoclast and osteoblast activity downstream [19,32,[60], [61], [62]]. Dysregulation of these pathways can convert physiological mechanical signals into catabolic and inflammatory responses, thereby contributing to pathological subchondral bone remodelling, cartilage degeneration and OA progression. Experimental evidence suggests that this sequence is particularly relevant during early disease, when mechanically induced alterations in subchondral bone turnover precede the development of subchondral sclerosis and extensive cartilage loss. In this context, PIEZO1 has been identified as a key regulator of mechanically induced Ca^2+^ signalling and catabolic gene expression in articular cartilage. Chondrocyte-specific deletion of Piezo1 resulted in attenuation of inflammatory and matrix-degrading responses and conferred significant protection against cartilage degeneration in an experimental OA model [62], providing causal evidence for its role in mechanotransduction-mediated OA progression. Further research showed that inflammatory conditions, as seen in OA, upregulate chondrocytic PIEZO1 expression, thereby increasing susceptibility to abnormal mechanical loading [61].

Research by Alliston and colleagues demonstrated that osteocytic TGF-β signalling is crucial for this mechanosensitive control of bone homeostasis and quality [63]. Specifically, osteocyte-specific loss of the TGF-β type II receptor led to abnormal subchondral bone thickening, increased sclerostin expression, and cartilage degeneration [31]. This renders subchondral bone unresponsive to physiological mechanical strain. Building on these pathway-level insights, the same group further demonstrated that osteocyte dysfunction at the perilacunar/canalicular remodelling (PLR) level drives OA-related changes. In human OA subchondral bone and in mice lacking osteocytic matrix metalloproteinase 13 (MMP13), suppression of PLR resulted in a disrupted osteocyte lacunocanalicular network, a hypermineralised and disorganised bone matrix, and compromised cartilage characterised by reduced proteoglycan content and altered chondrocyte anabolic–catabolic gene expression [32]. Together, these studies establish osteocytes as active mechanobiological regulators of subchondral bone, demonstrating that their TGF-β-dependent mechanosensing and MMP13-mediated PLR are necessary for maintaining a healthy bone microenvironment that supports cartilage homeostasis. Collectively, these findings support a hierarchical framework in which abnormal joint loading is sensed not only by chondrocytes but also osteocytes embedded in the subchondral bone matrix, leading to dysregulated subchondral bone remodelling, altered mechanical properties of the osteochondral unit, and ultimately cartilage degeneration. This positions osteocytic signalling dysfunction as a causal contributor to OA progression in experimental studies; however, the extent to which these mechanisms translate to human disease and can be therapeutically targeted remains to be fully clarified. These osteocyte-driven changes converge on the osteoclast-osteoblast uncoupling discussed next, mediated centrally by the RANK/RANKL/OPG axis.

### 4.3 RANK/RANKL/OPG signalling

Among the molecular pathways implicated in subchondral bone alterations in OA, the receptor activator of nuclear factor-κB (RANK), its ligand RANKL, and the decoy receptor osteoprotegerin (OPG) form a central regulatory axis controlling osteoclast differentiation and bone resorption. Dysregulation of RANK/RANKL/OPG signalling within the subchondral compartment has been implicated in abnormal bone remodelling in OA, including increased osteoclast activity, early trabecular thinning, and subsequent subchondral sclerosis in later disease stages [23,64,65]. Upton et al. demonstrated increased RANKL expression and osteoclast numbers in subchondral bone from hip and knee OA patients, which correlated with regions of cartilage loss in early to intermediate OA, supporting an association between osteoclast activation and early structural damage. Wang et al. showed that chondrocyte-derived OPG increased bone mass in a transgenic murine model, representing a possible pathophysiological mechanism underlying subchondral sclerosis in late-stage OA, although direct causality in humans remains unproven. Mechanical loading, inflammation, and crosstalk with chondrocytes and synovial cells further modulate RANK/RANKL/OPG signalling, thereby linking subchondral bone dynamics to overall joint degeneration. One emerging regulatory mechanism involves ubiquitin C-terminal hydrolase L1 (UCHL1), which is overexpressed in osteoclasts of osteoarthritic subchondral bone and appears to act as part of a negative feedback loop that limits osteoclast differentiation and activity. Liang et al. demonstrated that UCHL1 expression increased during OA progression in mice, and that UCHL1 overexpression suppressed RANKL-induced osteoclastogenesis *in vitro*, whereas UCHL1 knockdown enhanced osteoclast differentiation and resorptive capacity [66].

Osteocyte apoptosis, a process known to destabilise bone homeostasis, has been linked to increased RANKL expression. Earlier foundational work demonstrated that apoptotic osteocytes stimulate neighbouring viable osteocytes to upregulate RANKL, thereby driving osteoclast recruitment and activation [67,68] and providing mechanistic evidence for a causal upstream trigger of osteoclastogenesis. More recently, mechanotransduction pathways have gained attention, particularly the PIEZO1/yes-associated protein (YAP) axis. Mechanical strain sensed through the PIEZO1 ion channel activates YAP signalling, which has been shown to regulate RANKL/OPG balance [69]. Genetic deletion of *Piezo1* attenuated both cartilage degeneration and subchondral bone pathologies in a murine model [70], supporting a link between mechanotransduction and RANKL-mediated bone remodelling. Additionally, the immunoglobulin superfamily member 11 (IGSF11) - an adhesion molecule that acts as a co-stimulatory factor for osteoclast differentiation - has been found to be upregulated in osteoarthritic subchondral bone and to colocalise with RANK on osteoclast precursors. Kim et al. showed that IGSF11 enhanced RANKL-induced osteoclast maturation *in vitro* and that inhibition of IGSF11 reduced osteoclast formation, bone resorption and sclerosis of the subchondral bone plate in late-stage OA in an experimental model [21] (summarised in Fig. 3a).

**Fig. 3:**
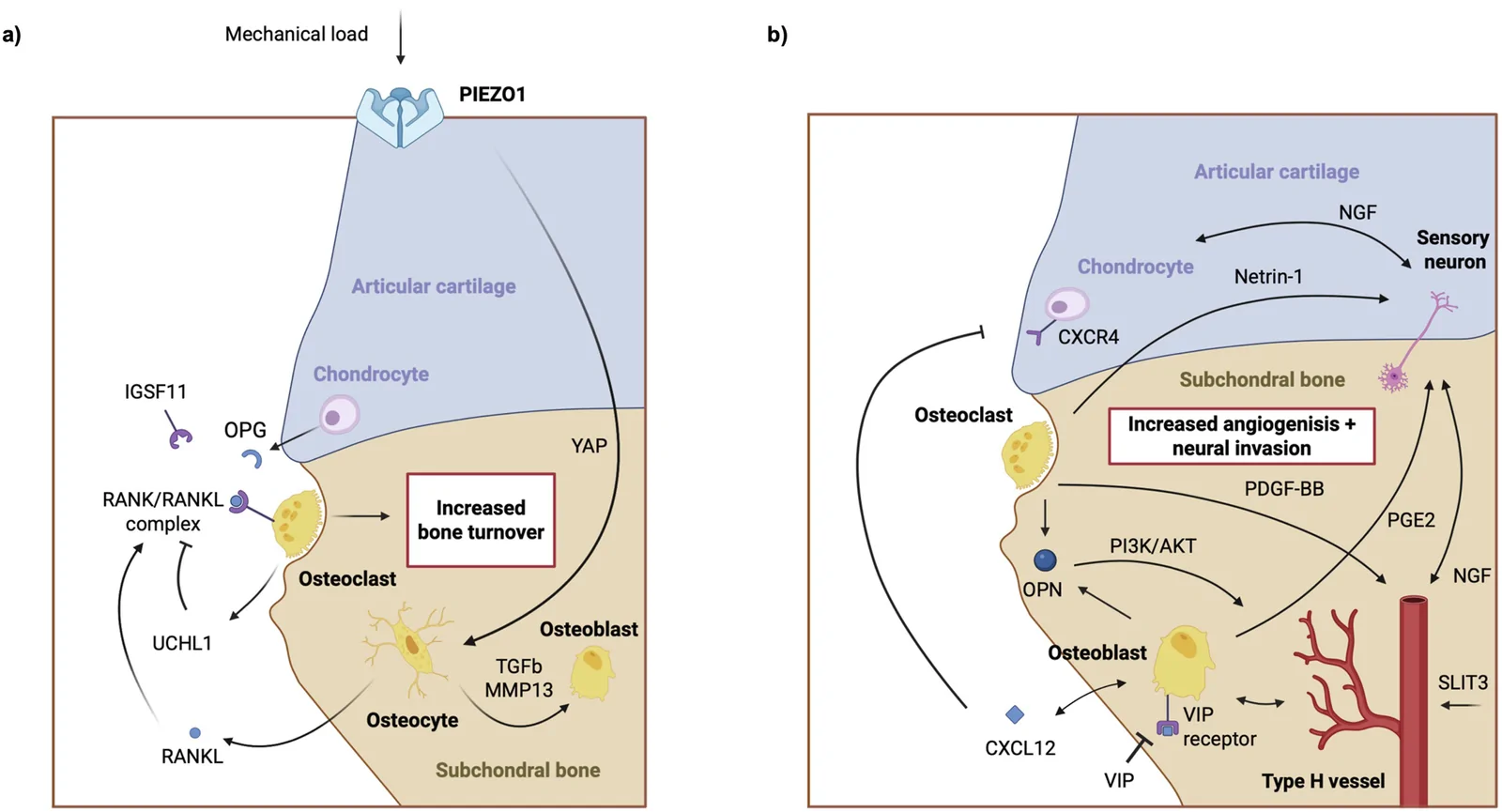
An overview of underlying molecular mechanisms, with emphasis on (a) RANK/RANKL/OPG signalling and (b) vascularisation and neurovascular coupling in the subchondral bone in OA.

Together, these findings highlight the complex regulation of RANK/RANKL/OPG-driven osteoclastogenesis, which represents a major factor contributing to pathological subchondral bone remodelling in OA, and has also been comprehensively reviewed elsewhere [71]. Sustained osteoclastic activity further promotes aberrant vascular invasion, a central feature of intermediate to late OA discussed in the following section.

### 4.4 Angiogenesis

Increasing evidence identifies aberrant angiogenesis within the subchondral compartment as a concomitant feature or consequence of dysregulated bone remodelling and a key contributor to OA development and progression. Neovascularisation promotes the ingrowth of sensory nerves into osteochondral tissues that are normally only sparsely innervated, and is increasingly recognised as a key factor in OA pain [5]. In this context, BMLs reflect focal regions of heightened subchondral bone turnover, angiogenesis, and inflammation, linking aberrant osteochondral remodelling to cartilage degeneration, pain, and accelerated structural OA progression [9]. Using dynamic contrast-enhanced MRI, de Vries et al. demonstrated that increased subchondral bone perfusion correlates with pain severity and radiographic OA progression [35], suggesting altered vascular dynamics as the disease advances. Vascular invasion from the subchondral bone into the normally avascular articular cartilage follows these remodelling events and disrupts tissue homeostasis, facilitates sensory nerve ingrowth, and accelerates matrix breakdown. Angiogenic signalling pathways - driven by mechanical stress, hypoxia, and inflammatory mediators – further promote subchondral bone remodelling and sclerosis, thereby perpetuating joint degeneration.

A major conceptual shift followed the identification of type H vessels - capillaries characterised by high CD31 and endomucin expression – which redefined the understanding of the coupling between angiogenesis and osteogenesis [72]. Kusumbe et al. showed that type H vessels directly regulate osteoprogenitor cell expansion and bone formation. Subsequent work confirmed that this specialised endothelial subset plays a central regulatory role in coupling vascular growth with osteoblast activity, as genetic ablation of the proangiogenic factor slit guidance ligand 3 (SLIT3) in mice decreased type H skeletal endothelium and significantly impaired osteogenesis and bone remodelling [73]. Importantly, type H vessel abundance is increased in the subchondral bone of OA patients and animal models, and precedes subchondral bone sclerosis [24], indicative of a potential upstream role in disease progression. Several intracellular pathways have been implicated in driving this aberrant angiogenic response, including phosphoinositide 3-kinase/protein kinase B (PI3K/AKT) signalling during early stages of OA [74]. This pathway appears to be modulated by osteopontin (OPN), as intraperitoneal injections of OPN increased type H vessel formation and subchondral bone sclerosis in a murine model, leading to increased endothelial proliferation, higher BV/TV, and accelerated cartilage degeneration [25]. *In vitro* studies using human umbilical vein endothelial cells (HUVECs) further demonstrate the complexity of this regulatory network, showing that subchondral bone sclerosis and type H vessel formation can be influenced by CXC motif chemokine ligand 12 (CXCL12) signalling, which is significantly elevated in murine OA knees and promotes endothelial migration and tube formation associated with subchondral sclerosis [26]. Similarly, vasoactive neuropeptides such as vasoactive intestinal peptide (VIP) modulate endothelial behaviour and angiogenic potential. Intraperitoneal administration of a VIP receptor antagonist alleviated subchondral bone sclerosis in OA mice [75], representing a potential pharmacological target for OA treatment, although evidence remains preclinical. Environmental oxygen levels also appear to influence disease progression, as induction of a hypoxic microenvironment was shown to slow OA progression in a murine model [76], indicating a link between angiogenesis, hypoxia-inducible pathways, and metabolic regulation.

Another major axis of regulation involves the endothelial platelet-derived growth factor-BB (PDGF-BB), which has been shown to promote angiogenesis in subchondral bone in experimental OA studies employing different transgenic mice [77,78]. An osteoclast-specific deletion of the E-type prostanoid receptor 4 (EP4) significantly reduced PDGF-BB production, type H vessel formation and bone sclerosis [79], supporting a mechanistic link between osteoclast activity and angiogenesis.

Therapeutically, antioxidant treatment has been shown to suppress excessive angiogenesis without notable adverse effects in rodent OA models. In this regard, Wu et al. reported reduced endothelial proliferation, inhibited osteoclast formation and bone resorption, and improved cartilage preservation upon treatment with antioxidant nanoparticles [80]. In addition, several natural anti-inflammatory compounds - including baicalein, betaine, and isoliquiritigenin - have been reported to exert protective effects against pathological vessel formation, as shown by reduced angiogenic marker expression and alleviated OA progression in different preclinical models [[81], [82], [83]]. Some repurposed pharmacological agents, such as antimalarial and antifungal drugs, have also been reported to attenuate aberrant angiogenesis in preclinical studies [84,85]. However, current evidence remains limited, comprising only a few preclinical models.

Collectively, these findings underscore the multifaceted regulation of angiogenesis in subchondral bone and highlight several promising molecular pathways that may be targeted in future therapeutic strategies, which aim to modify the disease rather than merely alleviating symptoms. Together, these vascular and remodelling changes converge on the osteochondral junction, increasingly recognised not as a passive boundary but as an active disease compartment in OA. Tidemark advancement and multiplication, calcified cartilage remodelling, and breaching of the subchondral bone plate enable bidirectional molecular exchange between bone and cartilage that would otherwise be blocked under physiological conditions. Vascular and neural invasion through this disrupted interface couples structural remodelling directly with pain generation, making the osteochondral junction a structural and biological hub linking subchondral bone pathology, cartilage degeneration, and clinical symptoms. Type H vessel ingrowth in particular is closely coupled with sensory neural invasion, linking the structural changes described here to OA-associated pain, which is examined in detail in the following section.

### 4.5 Neural invasion and pain generation

OA is closely linked to pain. Growing evidence indicates that pathological angiogenesis and sensory nerve ingrowth are closely coordinated processes within the osteochondral junction, linking vascular remodelling to pain generation, as recently reviewed elsewhere [86,87]. On a molecular level, different proteins and signalling molecules mediate neural invasion. Morphometric analyses of human OA knees have shown a strong correlation between endothelial cell proliferation and nerve growth factor (NGF) expression [88], suggesting a coupling of angiogenesis and neural invasion at the osteochondral junction (summarised in Fig. 3b). NGF is secreted by chondrocytes, fibroblasts and macrophages and promotes neuronal growth and survival in response to inflammatory stimuli such as IL-1β [89,90]. Accordingly, NGF expression has been shown to correlate with disease progression in human knee samples [29,30]. Its high-affinity receptor, tropomyosin receptor kinase A (TrkA), is expressed on sensory neurons, whereas the low-affinity receptor nerve growth factor receptor (NGFR), also known as p75 neurotrophin receptor, is expressed on chondrocytes. These findings provide the rationale for emerging experimental treatment strategies. Pharmacological blockade of TrkA reduced OA-associated pain in a mouse model; however, no effect on radiographic disease progression was observed [27]. Experimental knockout of NGF signalling reduced subchondral bone sclerosis and osteophyte formation [91], suggesting a potential structural role, although evidence remains preclinical. Tanezumab, a monoclonal antibody targeting NGF, improved pain and functional outcomes compared with nonsteroidal anti-inflammatory drugs (NSAID) treatment in patients with symptomatic hip and knee OA [92,93]. Notably, adverse effects such as rapid progressive OA or osteonecrosis occurred in a dose-dependent manner and more frequently in the treatment group, particularly when therapy was initiated in later disease stages [94], highlighting stage-dependent risk profiles which might limit clinical applicability.

Beyond neurovascular coupling, direct interactions of bone cells and nerves also contribute to OA pain. More recently, osteoclast-derived Netrin-1 has been shown to promote sensory nerve growth within the subchondral bone in rodent OA models [5], indicating a mechanistic link between bone resorption and pain generation. In addition, osteoblast-derived prostaglandin E2 (PGE2) increased the sensitivity of dorsal root ganglia to stimuli, while pharmacological inhibition of PGE2 reduced OA symptoms in a mouse model [95]. Together, these findings highlight that OA pain is driven by coordinated neurovascular and bone–nerve interactions, which may represent therapeutic targets for symptom alleviation, although their impact on structural disease progression remains uncertain. Beyond these established mechanistic axes, recent discoveries have expanded the molecular landscape of subchondral OA biology, as discussed below.

### 4.6 Emerging mechanisms and future directions

Recent studies have expanded our understanding of the molecular mechanisms driving subchondral bone alterations in OA. Emerging evidence highlights the contribution of osteocyte-derived extracellular vesicles (EVs) as mediators of intercellular communication at the osteochondral unit. These EVs transport bioactive molecules, such as non-coding microRNAs (miRNAs), which regulate gene expression via Wnt-regulated pathways [96,97] and represent an important epigenetic mechanism in OA. For example, miRNA-21 is upregulated in OA cartilage and inhibits chondrogenesis [98,99]. Furthermore, it mediates RANKL-induced osteoclastogenesis in a positive feedback loop [22] as demonstrated *in vitro*. In contrast, miRNA-148a exerts anabolic effects on cartilage, with overexpression leading to increased type II collagen deposition [100]. Further investigation of differentially expressed miRNA has enabled the establishment of regulatory networks in OA cartilage [101]. Unfortunately, attempts to use circulating miRNAs as OA biomarkers in the serum of affected patients have so far proven ineffective [102]. miRNAs also interact with cellular stress pathways. In this context, impaired chondrocyte autophagy has been described, indirectly affecting bone remodelling, whereas suppression of chondrocyte pyroptosis via miRNA-223 has been proposed as a potential treatment approach for OA, as summarised by Shang [103]. In parallel, the long non-coding RNA H19 has become a focus of research, as it has been shown to modulate TGF-β expression and influence inflammatory responses in chondrocytes. Recent preclinical work demonstrated that H19 levels in cartilage correlate with OA severity and promoted inflammatory gene expression in chondrocytes [104,105]. An osteocyte-specific deletion of H19 in mice alleviated OA progression, reduced subchondral bone sclerosis, and preserved cartilage structure [106]. Complementing these findings, studies on neurovascular regulation indicate that loss of the vasoactive neuropeptide calcitonin gene-related peptide alpha (αCGRP) exacerbates subchondral bone sclerosis in murine models [107]. Hildebrandt et al. demonstrated increased bone formation, angiogenesis, and cartilage damage in αCGRP-deficient OA mice. However, translation of CGRP-based interventions has so far been unsuccessful, as reflected by the premature termination of a related clinical trial [108].

Recent translational efforts targeting the subchondral bone have focussed on interventions at the osteochondral interface and on personalised approaches. Autologous and allogenic MSC-based therapies, delivered intra-articularly or directly into subchondral BMLs, have shown efficacy in delaying structural knee OA progression in early to intermediate disease stages, although long-term outcomes remain uncertain [109,110]. In parallel, preclinical gene-silencing approaches, such as intraarticular delivery of small interfering RNA targeting inflammatory signalling, have shown promising results in preclinical studies, as outlined by Grol [111]. Bone-specific targets, such as RANKL, have also been combined with RNA-based strategies. However, evidence remains limited to preclinical experimental approaches [112,113]. Advances in biomaterials have enabled the development of subchondral bone-targeted drug delivery systems, including nanocarriers engineered for bone affinity, as described below and summarised by Chen [114]. Furthermore, scaffolds are being investigated for their potential to enhance osteochondral regeneration. In rabbits and rats, three-dimensional-printed subchondral bone scaffolds improved subchondral bone regeneration and cartilage restoration in knee OA [115,116]. In patients with early-stage knee OA, implantation of a tri-layered scaffold at the osteochondral junction resulted in partial subchondral regeneration and improved clinical symptoms [117], although evidence remains limited to early-phase studies.

Together, these broad approaches highlight a shift toward localised, mechanism-driven therapies that directly address subchondral pathology to potentially modify OA progression (summarised in Table 2). However, it is crucial to determine the therapeutic efficacy of these approaches with regard to the location, stage and subchondral bone endotype of OA. Translation into routine clinical practice remains limited as a result, and further studies are required to establish efficacy, safety, and patient stratification strategies.

**Table 2:** Therapeutic targeting of subchondral bone pathways in OA: mechanisms and evidence.

Targeted pathway	Drugs	Evidence Type	Joint and OA type	Key findings	Evidence level	Limitations
**Systemic metabolic modulation**	Semaglutide (GLP-1 agonist)	Clinical + preclinical	Mild to moderate knee OA in obese patients	Pain reduction; unclear structural effects; preclinical data suggest structural benefit [58,59]	Moderate (symptoms), speculative (structure)	Confounding by weight loss; no structural endpoints
**Neurovascular modulation**	VIP agonists	Preclinical	OA mouse models	Reduced subchondral sclerosis [75]	Speculative	Preclinical only
**Angiogenesis**	Antioxidant nanoparticles Antimalarial/antifungal drugs	Preclinical	OA rodent models	Reduced angiogenesis, bone resorption, improved cartilage [80] Reduced aberrant angiogenesis [84,85]	Speculative	Preclinical only
**Inflammation**	Natural compounds (baicalein, betaine, isoliquiritigenin)	Preclinical	OA rodent models	Reduced angiogenesis and OA progression [[81], [82], [83]]	Speculative	Limited translational relevance
**Pain**	Tanezumab (anti-NGF)	Clinical	Symptomatic hip and knee OA	Pain reduction, no structural benefit; risk of rapid OA progression [[92], [93], [94]]	Moderate (symptom control)	Safety concerns; stage-dependent adverse effects
**Gene silencing**	miRNAs	Preclinical	OA rodent models	Reduced OA progression [112,113]	Speculative	Preclinical only
**Regenerative/immunomodulatory**	MSC-based therapies	Clinical + preclinical	Early to intermediate knee OA	Delay of structural progression [109,110]	Moderate	Heterogeneous protocols; long-term effects unclear
**Structural regeneration**	Osteochondral scaffolds	Preclinical + early clinical	Early knee OA	Improved subchondral regeneration and symptoms [[115], [116], [117]]	Moderate	Limited long-term data

## 5 Drug interactions with subchondral bone

### 5.1 Antiresorptive drugs

Based on the proposed role of the subchondral bone compartment in OA, the repurposing of established osteoporosis drugs for OA appears as an attractive strategy [118]. Stratifying patients by subchondral bone phenotype and disease stage, and differentiating between antiresorptive and anabolic osteoporosis therapies, may enable improved therapeutic outcomes in selected patient subgroups. Bisphosphonates are widely used antiresorptive agents for the treatment of osteoporosis, as they inhibit osteoclast differentiation and activity, thereby reducing bone turnover. Given the established involvement of subchondral bone remodelling in the pathogenesis of OA, these agents have been explored as potential disease-modifying therapies.

Preclinical studies have demonstrated beneficial effects of bisphosphonates on joint integrity, particularly in early disease stages characterised by high bone turnover. Alendronate, a bisphosphonate commonly prescribed for osteoporosis, was shown to attenuate joint degeneration in a murine post-traumatic OA model when administered immediately after injury [119]. In this study, early alendronate treatment suppressed injury-induced osteoclast activation in the subchondral bone, leading to reduced bone resorption, preservation of subchondral microarchitecture, and subsequent protection of overlying articular cartilage. Additional rodent studies have reported protective effects of alendronate during early OA, including reduced subchondral bone sclerosis, decreased osteoclast activity, and preservation of cartilage structure [[120], [121], [122]]. While Khorasani et al. demonstrated that alendronate treatment preserved trabecular bone structure by inhibiting excessive osteoclast-mediated bone turnover, Lambova et al. further showed that alendronate reduced subchondral bone sclerosis and decreased expression of cartilage degradation markers, resulting in preservation of cartilage integrity and reduced osteophyte formation. Feng et al. reported that early alendronate treatment limited aberrant bone–cartilage crosstalk, thereby preventing progressive cartilage matrix breakdown. These findings suggest that early antiresorptive intervention may preserve bone compliance and prevent maladaptive increases in stiffness that occur in later stages. Despite this strong preclinical support, human evidence remains limited. In a clinical trial involving patients with hip OA, administration of 35 mg alendronate once weekly resulted in significant pain reduction and reduced urinary bone turnover markers N-telopeptide of type I collagen (NTX-I) and C-telopeptide of type II collagen (CTX-II). However, the treatment did not alter structural disease progression [123], indicating that pharmacological inhibition of bone turnover does not necessarily translate into structural modification in cases with manifest disease. In combination with nanoparticle systems, alendronate-conjugations have recently been investigated in preclinical models of osteoporosis [124,125], and may represent a strategy to target subchondral bone in OA, although this remains speculative. In sum, preclinical evidence strongly indicates beneficial effects of alendronate in OA, whereas clinical data remains scarce and inconclusive.

Other bisphosphonates, such as zoledronic acid - a more potent, intravenously administered agent - have demonstrated dose- and time-dependent protective effects in various preclinical OA models [[126], [127], [128]]. She et al. showed that zoledronic acid suppressed osteoclast-driven subchondral bone remodelling, reduced bone microdamage, and delayed cartilage erosion in surgically induced OA in rabbits. Xu et al. further reported stabilisation of subchondral bone microarchitecture in a canine model. In a rodent shoulder OA model, Tawaratsumida et al. demonstrated that zoledronic acid reduced inflammatory signalling and cartilage deterioration by suppression of synovial M1 macrophages and osteoclast numbers, leading to improved joint congruency and cartilage preservation. These findings are consistent with the ability of potent bisphosphonates to suppress aberrant subchondral bone turnover, reduce BMLs, and potentially modulate pathological osteochondral crosstalk. However, clinical studies have shown conflicting results. A recent meta-analysis of randomised clinical trials concluded that bisphosphonates provide no consistent clinical benefit in terms of knee OA symptoms or radiographic disease progression [129], suggesting that heterogeneity in patient populations, OA severity, joint type, and timing of treatment initiation and/or duration may obscure potential subgroup-specific effects.

Denosumab, a RANKL-neutralizing antibody, potently suppresses osteoclast formation and activity. *In vitro* studies have shown that denosumab reduces osteoclastic bone resorption in subchondral bone tissue derived from murine OA knees [130], supporting a direct effect on bone turnover. However, clinical evidence on its efficacy in OA is scarce. A randomised, placebo-controlled Phase 2a trial showed that denosumab reduced progression of erosive hand OA over 24–48 weeks, indicating that RANKL-mediated bone resorption contributes to structural damage in this OA subtype characterised by high bone turnover and erosive features [131]. Importantly, these positive findings cannot be generalised, as verification in other OA types and joints is lacking up to date.

Strontium ranelate acts as a dual bone agent: it inhibits osteoclastic bone resorption and increases osteoblastic bone formation. In a canine OA model, strontium ranelate reduced subchondral bone thickening and decreased cartilage degradation [132]. In contrast, increased BMD and trabecular thickness of the subchondral bone was observed in a different study employing guinea pigs [133]. Similar results were obtained in a preclinical study employing rats. However, in this model, only high doses of strontium ranelate led to osteo- and chondroprotective effects, as shown by significantly reduced cartilage matrix loss and inhibition of subchondral bone loss [134], suggesting a dose-dependent modulation of bone remodelling. Mechanistic studies revealed that the observed effects may be mediated by inhibition of osteoblastic MMP-2 and MMP-9 expression, which in turn limits extracellular matrix breakdown at the osteochondral interface and hence stabilises both bone and cartilage compartments [135]. Several experimental studies further reported that strontium ranelate protects against cartilage damage and reduces pain in OA [136,137], indicating that modulation of bone remodelling may indirectly influence nociceptive pathways. In a double-blind, randomised placebo-controlled clinical trial, daily administration of 1-2 g strontium ranelate reduced radiographic knee OA progression with a reduction in joint space narrowing and improved symptoms [138]. Similarly, long-term treatment was associated with BML reduction and slower structural progression [139,140]. Due to cardiovascular side effects, however, the agent was withdrawn from markets worldwide in 2017 and is no longer available as an anti-osteoporotic drug [141,142]. Re-evaluation in OA would require resolution of those safety concerns, which to date has not been demonstrated; the drug therefore stands as an example of bone-targeted efficacy in OA that could not be translated under current safety standards.

In sum, antiresorptive therapies may be most effective in early and or high-turnover (e.g., erosive) stages or forms of OA, whereas their impact in later sclerotic stages seems limited.

### 5.2 Anabolic agents

Teriparatide, a recombinant human parathyroid hormone analogue (PTH 1–34), is an osteoanabolic agent that stimulates osteoblast proliferation and activity, thereby increasing bone formation and improving bone microarchitecture. Because alterations in subchondral bone structure contribute to OA progression, teriparatide has been investigated for its potential to modulate osteochondral remodelling. Preclinical studies have demonstrated protective effects of teriparatide on subchondral bone in OA. In a mouse model of injury-induced OA, systemic teriparatide inhibited aberrant chondrocyte maturation, reduced cartilage degeneration, and promoted cartilage matrix regeneration [143]. In rabbits, intermittent PTH injections improved subchondral bone microarchitecture and reduced cartilage degeneration, as indicated by increased trabecular thickness and connectivity in the subchondral bone as well as reduced cartilage fibrillation and proteoglycan loss [18]. Similar outcomes were reported in guinea pigs, where teriparatide preserved subchondral trabecular structure, increased BV/TV and BMD in the subchondral bone, reduced osteoclast activity and increased the OPG/RANKL ratio in the cartilage [144], indicating a shift toward a more balanced bone remodelling state. In mice, administration of teriparatide immediately after knee injury increased subchondral trabecular bone volume and alleviated cartilage degeneration. Delayed treatment after 8 weeks similarly increased bone volume but was associated with elevated expression of type I collagen in subchondral bone, indicating ongoing bone formation even at later disease stages, where subchondral bone loss is usually observed [143]. This suggests that anabolic therapy could also contribute to increased stiffness in advanced OA, depending on timing and local remodelling dynamics. Mechanistically, teriparatide has been shown to act via pathways that promote osteoblast differentiation and survival, as preservation of subchondral architecture has been linked to activation of the janus kinase 2/signal transducer and activator of transcription 3 (JAK2/STAT3) signalling cascade. Pharmacological inhibition of the latter reversed the bone-protective effects of teriparatide in a murine OA model [145], providing causal evidence in experimental systems. However, the fact that the PTH receptor is upregulated in chondrocytes suggests that it may also have a direct effect on articular cartilage beyond its effects on subchondral bone [143]. In contrast to the growing body of preclinical work, human data remain lacking. A registered clinical trial evaluating teriparatide in knee OA (ClinicalTrials.gov ID: NCT03072147) has not yet reported results. Thus, while experimental models suggest that teriparatide can beneficially modulate subchondral bone remodelling and slow structural deterioration in OA, its therapeutic potential in humans remains unclear.

Romosozumab, a monoclonal antibody against sclerostin, exerts a dual action by stimulating bone formation and reducing bone resorption. However, its effects in OA appear limited. In women with coexisting osteoporosis and knee OA, romosozumab treatment did not significantly affect pain, functional outcomes, or radiographic OA progression, despite increasing systemic BMD [146]. Likewise, a comparative analysis reported that neither denosumab nor romosozumab altered osteophyte development in the knee joint [147], suggesting that systemic modulation of bone turnover does not necessarily translate into local structural benefits in OA (summarised in Table 3). As both agents have been introduced into clinical practice in the more recent past, their potential role as disease-modifying treatments for OA is still the subject of research.

**Table 3:** Overview of different anti-osteoporotic drug classes and their effects on the subchondral bone in OA.

Drug	Effects on the subchondral bone	Study type	OA type	Study endpoints	Limitations
**Antiresorptive drugs**					
Alendronate					
	Preserves subchondral bone volume, inhibits osteoclast activation [119,120]	Preclinical rodent model	Posttraumatic knee OA	Structural outcome	Early treatment initiation required
	Reduces subchondral bone sclerosis [122]	Preclinical rodent model	Collagenase-induced knee OA	Cartilage biomarkers	No structural outcome assessed
	Improves microstructure of the subchondral bone with increased trabecular markers [121]	Translational (rodent model + prospective clinical trial)	Posttraumatic knee OA (rodent), knee OA (human)	Structural outcome (rodent), pain scores (human)	Small clinical cohort (n = 30)
	No effects on structural disease progression [123]	Prospective clinical trial	Hip OA	Structural outcome, pain scores	Small clinical cohort (n = 50)
Zoledronic acid					
	Increases subchondral bone density [126,127]	Preclinical rodent model	Posttraumatic knee OA	Structural outcome	Dose- and time dependent effects
	Decreases RANKL/OPG expression ratio and number of osteoclasts [128]	Preclinical rodent model	Rotator cuff arthropathy	Structural outcome	Early treatment initiation required
	No effects on disease progression [148]	Prospective clinical trial	Knee OA	Structural outcome, pain scores	Moderate clinical cohort (n = 190)
	Reduces bone marrow lesions in knee OA after 6 months [149]	Prospective clinical trial	Knee OA	Structural outcome	Small clinical cohort (n =59); conflicting RCT data
Denosumab					
	Inhibits RANKL-induced osteoclastogenesis and hence bone resorption [130]	Prospective rodent model	Posttraumatic knee OA	Structural outcome	Early treatment initiation required
	Reduces progression of erosive hand OA [131]	Prospective clinical trial	Hand OA	Structural outcome	Results limited to hand OA
Strontium ranelate					
	Reduces subchondral bone sclerosis [132]	Preclinical canine model	Posttraumatic knee OA	Structural outcome, serum biomarkers	Ethically controversial model
	Increases BMD in the subchondral bone [133,136]	Preclinical rodent model	Posttraumatic knee OA	Structural outcome	Cross-sectional studies
	Decreases joint space narrowing and BMLs in knee OA [[138], [139], [140]]	Prospective clinical trial	Knee OA	Structural outcome, pain scores	Safety concerns in human use
**Anabolic agents**					
Teriparatide					
	Improves subchondral bone microstructure [18]	Preclinical rodent model	Posttraumatic knee OA	Structural outcome	Preclinical evidence only
	Increases BMD in the subchondral bone in a dose-dependent manner [144]	Preclinical rodent model	Age-related knee OA	Structural outcome	Preclinical evidence only
	Promotes osteoblast differentiation and survival [145]	Preclinical rodent model	Collagenase-induced knee OA	Structural outcome	Preclinical evidence only
	Increases expression of *Col1a1*, preventing subchondral bone loss [143]	Preclinical rodent model	Posttraumatic knee OA	Structural outcome	Dose-dependent effects
Romosozumab					
	No effects on disease progression in patients with knee OA [146,147]	Prospective clinical trial	Knee OA	Structural outcome, pain scores	Possible placebo response in pain scores

### 5.3 Translational failure of therapies modulating subchondral bone metabolism

Despite encouraging results in preclinical studies, most bone-targeted therapies have not yet been incorporated into routine clinical management of OA. Several factors may explain this translational gap. First, experimental OA models differ substantially in their underlying pathophysiology, as reviewed elsewhere [150]. Commonly used surgically induced instability models may overestimate the efficacy of bone-targeted interventions compared with the heterogeneous clinical manifestations of OA [151] and therefore limit translational applicability. Second, successful modulation of structural bone remodelling does not necessarily translate into clinically meaningful improvements in pain or physical function, which represent distinct therapeutic endpoints. Finally, safety considerations have limited clinical implementation of otherwise promising agents, exemplified by the withdrawal of strontium ranelate because of cardiovascular adverse events. Importantly, treatment response may also vary between OA phenotypes and disease progression. Early OA stages are associated with increased osteoclast activity, suggesting that anti-resorptive therapies such as denosumab may be more effective when administered early. However, most clinical trials are conducted in later-stage OA, and therefore potentially miss this therapeutic window. Moreover, it remains unclear whether the observed positive effects of denosumab on erosive hand OA can be transferred to other OA phenotypes and joints, which exhibit distinct biomechanical and inflammatory characteristics. Morphologically, hand OA shares features with rheumatoid arthritis, such as synovial inflammation and bone erosions, whereas knee and hip OA are more strongly influenced by mechanical loading. Therefore, therapeutic effects need to be evaluated in a context- and joint-specific manner. Additional preclinical studies and well-designed clinical trials are needed to clarify whether targeted antibody-based regulation of bone turnover can meaningfully influence subchondral bone health in different OA locations, particularly in early disease stages where bone remodelling is most dynamic [152].

Collectively, clinical trials of bone-targeting agents in OA have produced heterogeneous results. A biomarker- and imaging-informed stratification framework could therefore be further explored and refined to improve trial design by enriching for patients with a “bone-driven” OA phenotype and by aligning endpoints with the targeted mechanism (e.g., BML volume, perfusion metrics, and bone turnover markers) [153]. Such an approach may also clarify therapeutic windows in which antiresorptive or osteoanabolic interventions are most likely to influence osteochondral remodelling rather than merely modifying systemic bone density. Endotype-informed stratification, for instance distinguishing low-turnover from high-turnover OA subgroups, could prospectively identify patients most likely to benefit from antiresorptive versus osteoanabolic interventions, although prospective validation in interventional trials is required. Candidate biomarkers including serum CTX, procollagen type I N-propeptide (P1NP), urinary NTX-I, and CTX-II may serve as eligibility criteria for trials enriching for high-turnover phenotypes, although validated cutoffs distinguishing OA-relevant from osteoporosis-relevant ranges have yet to be established, as recently reviewed by Hannai et al. [154]. Future trials should also pre-specify which endpoint a given agent is expected to influence: symptomatic relief (e.g., WOMAC pain scores), imaging improvement (e.g., BML volume reduction), or true structural modification (e.g., joint-space narrowing, cartilage volume). These endpoints are not interchangeable, and conflating them has hampered interpretation of past clinical trials.

## 6 Conclusion

Over the past decade, substantial advances have broadened our understanding of the role of the subchondral bone in the initiation and progression of OA. Once considered a passive responder to cartilage degeneration, the subchondral bone is now recognised as a dynamic and active participant in joint homeostasis and disease pathology. Anatomical and imaging studies have highlighted the complexity of the osteochondral unit and identified characteristic morphological changes - such as altered bone turnover, sclerosis, cyst formation, and remodelling of both trabecular and cortical compartments - that are associated with symptomatic and structural progression of OA. At the molecular level, recent findings demonstrate that dysregulated crosstalk among osteoblasts, osteoclasts, osteocytes, chondrocytes, sensory nerve fibres, and immune cells, together with pathological neoangiogenesis, contributes to pathological remodelling of the subchondral microenvironment. While several experimental studies support causal roles for specific pathways, much of the current evidence in humans remains associative. Concurrently, high-resolution profiling technologies are transforming the understanding of subchondral bone biology. Single-cell RNA sequencing and spatial transcriptomics have revealed previously unrecognized heterogeneity among osteoblast and osteocyte populations, as well as distinct pathological cell states associated with OA progression. Spatially resolved omics further enable mapping of molecular gradients across the osteochondral unit, providing insights into bone–cartilage crosstalk and coordination of inflammatory and remodelling signals, and might even provide the basis for therapeutic subtypes, so called theratypes, as recently reviewed elsewhere [155]. Moreover, emerging cross-tissue atlas efforts integrating bone, cartilage, and synovium datasets are beginning to define OA endotypes and patient-specific molecular signatures, although their clinical applicability remains under investigation. Preclinical studies investigating osteoporosis-related pharmacotherapies further underscore the therapeutic relevance of subchondral bone. Both anti-resorptive and anabolic agents have selectively demonstrated the capacity to modulate bone remodelling, with variable downstream effects on cartilage integrity and clinical symptoms, although their efficacy appears to depend on disease stage, treatment timing, dosage, and the underlying biological OA phenotype, and remains inconsistent across clinical studies. These findings support a conceptual shift from OA as a uniform degenerative disease toward a heterogeneous joint disorder, characterised by spatially and biologically distinct subtypes. The relative contributions of mechanical loading, metabolic dysregulation, inflammation, and bone remodelling likely differ between joints, disease stages, and patient subgroups, with implications for therapeutic responsiveness. To address this heterogeneity, future clinical trials will require improved patient stratification, including imaging-informed enrolment strategies and as well as the integration of validated biomarkers combined with clinical and radiographic endpoints. Such approaches may enable identification of patient populations most likely to benefit from bone-targeted therapies and help define stage-specific therapeutic windows.

## Contributions

Conceptualization, A.D., J.K.; writing—original draft preparation, A.D., W.X., J.K.; writing—review and editing, A.D., W.X., A-R.A., A.B., T.R., J.K.; visualization, A.D., supervision, T.R., J.K. All authors have read and agreed to the published version of the manuscript.

## Declaration of generative AI in scientific writing

During the preparation of this work, the authors used Chat-GPT-5 to improve readability and to structure and summarise complex information, particularly in the abstract, the introduction and the conclusion. Smaller modifications (e.g. phrasing, grammar) have been made throughout the whole manuscript. No thematic content or scientific sources have been derived from Chat-GPT. All authors have read the manuscript thoroughly before submission and verified the AI-generated wording for accuracy. Modifications have been performed as needed. No other generative AI or AI-assisted technologies have been used to write the manuscript.

## Funding

AB, TR, and JK are supported by the 10.13039/501100001659German Research Foundation (10.13039/501100001659DFG) within the Clinical Research Unit *ProBone* under the grant numbers BA 7863/1-1 (to AB), RO 5925/5-1 (to TR), and KE 2179/11-1 (to JK).

## Declaration of competing interest

The authors declare no competing interests.
