# Supplementary material for: The emerging role of subchondral bone homeostasis in osteoarthritis
Source: J Orthop Translat. 2026 Oct 13;61:101233. doi: 10.1016/j.jot.2026.101233 (PMC13681461; doi:10.1016/j.jot.2026.101233)
Supplement: Supplementary file 1 [file mmc1.docx]

| **Joint site** | **Disease drivers** | **Subchondral bone response** |
| --- | --- | --- |
| **Hip** | Mechanical (cam morphology, dysplasia, axis malalignment)  Post-traumatic injury  Subchondral endotype heterogeneity | Variable bone mass with endotype-dependent low- or high-turnover patterns; osteocyte-driven remodelling; heterogeneous mineralisation; BMAT-osteoblast crosstalk |
| **Knee** | Mechanical overload  Varus/valgus malalignment  Post-traumatic injury  Metabolic inflammation | Early osteoclast-driven resorption; BML formation; type H vessel angiogenesis; late-stage sclerosis |
| **Hand** | Inflammatory / autoimmune-adjacent processes  Hormonal contribution (e.g. postmenopausal predominance) | RANKL-mediated osteoclastogenesis; erosive bone changes; synovial inflammation overlapping with rheumatoid features |
| **Shoulder** | Rotator cuff insufficiency  Chronic instability | Increased osteoclast activity; inflammatory subchondral remodelling; cartilage deterioration; secondary sclerosis |
| **Spine** | Mechanical / axial loading  Ageing | Osteophyte formation via endochondral ossification; vertebral endplate sclerosis; disc-space narrowing |

**Supplementary Table 1.** A joint-specific and endotype-oriented framework of OA.
